# The burden, risk factors, and antimicrobial susceptibility pattern associated with extended-spectrum beta-lactamase-producing *E. coli* and *K. pneumoniae* carriage among neonates and their surroundings at a referral hospital in the Moshi municipality

**DOI:** 10.3389/frabi.2025.1556842

**Published:** 2025-05-21

**Authors:** Happyness J. Mshana, Dorottya Kovacs, Florida Muro, Ruth Zadoks, Katarina Oravcova, Louise Matthews, Blandina T. Mmbaga

**Affiliations:** ^1^ Department of Research and Clinical Trial, Kilimanjaro Clinical Research Institute, Moshi, Tanzania; ^2^ Boyd Orr Centre for Population and Ecosystem Health, School of Biodiversity, One Health and Veterinary Medicine, College of Medical, Veterinary and Life Sciences, University of Glasgow, Glasgow, United Kingdom; ^3^ Paediatric Department, Kilimanjaro Christian Medical Centre, Moshi, Tanzania; ^4^ Paediatric and Child Health Department, KCMC University, Moshi, Tanzania; ^5^ Sydney School of Veterinary Science, Faculty of Science, University of Sydney, Camden, NSW, Australia

**Keywords:** burden, risk factor, antimicrobial susceptibility, extended-spectrum beta-lactamase (ESBL), neonates

## Abstract

**Introduction:**

Infections are a major driver of broad-spectrum antibiotic use. This wide use of antibiotics contributes to the emergence of antimicrobial resistance globally that poses a threat to human and animal health. Infections continue to be a major cause of death among pregnant women and neonates. Therefore, this study aimed to assess the burden of extended-spectrum beta-lactamase (ESBL)-producing *E. coli* and *K. pneumoniae* carriage among neonates and their surroundings admitted to a referral hospital in Northeast Tanzania.

**Methodology:**

The burden of ESBL-producing *E. coli* and *K. pneumoniae* in a neonatal ward was assessed by screening neonates’ rectums, maternal and healthcare workers’ hands, and neonatal cots. Isolates were cultured, identified, and tested for antimicrobial resistance, while generalized linear models identified risk factors for carriage.

**Results:**

A total of 437 neonates were screened for ESBL-producing *E. coli* and *K. pneumoniae*, with 235 (54%) being male. In addition, 77 maternal hand swabs, 118 neonatal cots, and 45 healthcare workers’ hand swabs were collected. ESBL-producing *K. pneumoniae* was isolated from 198 neonates (45%), and *E. coli* from 96 (23%). Additionally, 5% of maternal hands and 22% of neonatal cots were contaminated with these resistant bacteria. Overall ampicillin resistance was frequent in ESBL-producing *E. coli* and ESBL *K. pneumoniae* neonatal colonization (n=261,100%), as was resistance to trimethoprim-sulfamethoxazole (*n* = 233,89%), gentamicin (*n* = 169, 66%), and tetracycline (*n* = 140,54%). Only three (1%) of the ESBL-producing *E. coli* and ESBL *K. pneumoniae* isolates were resistant to meropenem. Risk factors significantly associated with carriage of either ESBL-producing *E. coli* or *K. pneumoniae* were being born in an admission room [odds ratio (OR)=1.95, confidence interval (CI)=1.31-3.13, p=0.006] and delivery mode, with vaginal delivery associated with a reduced risk of carriage (OR=0.57, CI=0.35–0.92, p=0.023).

**Conclusion:**

The study reveals a high burden of ESBL-producing *K. pneumoniae* and *E. coli* in neonates and their environment, with frequent resistance to ampicillin and gentamicin. Hospital admission and cesarean delivery increase the risk of carriage, while vaginal delivery lowers it. Active screening upon admission and advanced diagnostic methods can help reduce transmission and guide effective antimicrobial treatment.

## Introduction

Antimicrobial resistance (AMR) occurs when bacteria, viruses, fungi, and parasites no longer respond to treatment, making infections harder to treat and also increasing the risk of transmission, severe illness, and death (World Health Organization, 2015). The global prevalence of extended-spectrum beta-lactamase (ESBL)-producing *Enterobacteriaceae* (EPE) is increasing (Raphael et al., 2021). In East Africa, EPE prevalence ranges from 6% to 17% and 38% to 83% in community and hospital settings, respectively. In Tanzania, different studies have reported the prevalence of fecal carriage of EPE to be 50% among children admitted to a tertiary hospital and 12% among healthy community children (Tellevik et al., 2016). ESBL-producing *Escherichia coli* and *Klebsiella pneumoniae* are major multidrug-resistant bacteria that are known to cause serious hospital and community-acquired infections worldwide (Teklu et al., 2019; Husna et al., 2023). Infections such as sepsis, urinary tract infections, and diarrhea are major drivers of broad-spectrum antibiotic use. These contribute to the emerging global threat of antimicrobial resistance and decrease the effectiveness of first-line antimicrobial therapies.

Global efforts to tackle the threat of AMR have been discussed at the highest level, including the World Health Assemblies in 2014, 2015, and 2019, and the United Nations General Assembly in 2016 (World Health Organization, 2015). In 2015, the General Assembly of the World Health Organization (WHO) adopted a global action plan on AMR in recognition of the immediate threat AMR poses to human and animal health, productivity, and prosperity. The WHO urged its Member States to develop National Action Plans (NAPs) for AMR. In 2017, Tanzania adopted its NAP for 2017–2022, recognizing the need for action (Ministry of Health, n.d.). However, despite the existence of regulation and training programs, adherence to the guidelines does not always take place accordingly, and therefore antimicrobial resistance is accelerating. In Tanzania, antimicrobial resistance is a major problem and there are high levels of inappropriate use of antimicrobials in the human and animal sectors (Sangeda et al., 2021). There is, however, no process for systematically collecting data on the prevalence of antibiotic resistance in common pathogens. One of the objectives of NAPs is to strengthen the knowledge and evidence through surveillance and research, which are important for infection prevention and control measures, reducing the risk of transmission and acquisition of bacteria (Ministry of Health, n.d.). Therefore, this study aimed to address gaps identified in Tanzania’s National Action Plan to Combat Antimicrobial Resistance, including the lack of national surveillance for pathogens such as *E. coli* and *K. pneumoniae* and the absence of a systematic data collection approach for effective infection control, by assessing the burden, risk factors, and antimicrobial susceptibility patterns associated with the carriage of ESBL-producing *E. coli* and *K. pneumoniae* among neonates, and in their surroundings, at a referral hospital in north-eastern Tanzania.

## Methods

### Study design and area

This was a cross-sectional study conducted at the neonatal ward at a referral hospital, Kilimanjaro Christian Medical Center, in the Kilimanjaro region of Tanzania. The neonatal ward has a total of 45 neonatal cots, 3 incubators, and 18 beds. The majority of the neonates are born at the facility while others are referred from other facilities or born at home. The majority of caretakers/mothers practice subsistence farming, livestock keeping, and small businesses.

### Sample size

The sample size for active surveillance (measuring within-hospital transmission of *E. coli* in neonatal wards) was calculated based on the expected prevalence of ESBL-producing coliforms (25% of rectal swabs), a colonization period of approximately 1 week, and a Poisson distribution for the size of patient clusters colonized by ESBL-producing coliforms. To gather sufficient data on the actual cluster size, which reflects the risk of colonization with ESBL-producing coliforms in the neonatal ward, we aimed to collect samples from 330 neonates.

### Sample collection

Samples were collected between 2019 and 2021 across different seasons from the neonates at the neonatal ward after admission and consent was obtained. The exact start date was August to November 2019 for the first round, and the second round was from August to November 2021.

A sterile swab was gently inserted into the neonate’s anal sphincter. The sample was then stored in a sterile container with Amies transport medium and labeled with the patient’s identification number and date of collection. The specimen was then transported to the laboratory and processed on the same day. Swabs of maternal hands, healthcare workers’ hands, and neonatal cots were also collected and transported to the laboratory for microbiological processing.

### Laboratory procedures

Neonatal rectal, hand, and bed swabs were inoculated onto MacConkey agar supplemented with cefotaxime 2 μg/ml for screening of ESBL-producing organisms. *E. coli* and *K. pneumoniae* were phenotypically identified based on lactose fermentation on MacConkey agar, urease negativity, Simmons citrate negativity, sulfur indole motility test results (i.e., no production of hydrogen sulfide gas, indole positivity, and motility), and the fermentation on triple sugar iron test. AST was done on Mueller Hinton agar using the Clinical Laboratory Standard Institute (CLSI) guideline.

### ESBL and antibiotic susceptibility testing

Cefotaxime-resistant *E. coli* and *K. pneumoniae* isolates were screened for ESBL production with the combined diffusion method using ceftazidime (30 µg), cefotaxime (30 µg), and amoxicillin/clavulanate (30 µg discs) (Oxoid Ltd) as the inhibitory substances. The test was considered positive for ESBL when there was a synergy between any two antibiotics with amoxicillin/clavulanate. Susceptibility to 11 antibiotics was tested. Specifically, the disks used were ampicillin (10 μg), trimethoprim-sulfamethoxazole (1.25/23.75 μg), amoxicillin-clavulanic acid (20/10 μg), ciprofloxacin (5 μg), gentamicin (10 μg), piperacillin-tazobactam (100/10 μg), ceftriaxone (30 μg), ceftazidime (30 μg), and meropenem (10 μg). Confirmation of ESBL carriage was done using the combined disk diffusion method. The results of the susceptibility test were interpreted as sensitive, intermediate, and resistant based on the CLSI guideline (Weinstein and Clinical and Laboratory Standards Institute (CLSI), 2018). The control strains used were *E. coli* ATCC 25922, and *K. pneumoniae* ATCC 8868.

### Statistics

Data from the questionnaires were entered using RedCap (https://project-redcap.org/), downloaded to MS Excel, and analyzed using the analysis tool pack software. Descriptive statistics were used to summarize the data, with frequencies and percentages reported for categorical variables, and measures of central tendency and their dispersion for numerical data. The Chi-square test was used to conduct an initial univariable analysis of the association between potential risk factors and ESBL-producing *E. coli* and *K. pneumoniae* carriage.

We also conducted a multivariable analysis using generalized linear models (GLMs) in R (R Core Team, 2023) with a binomial family. The outcome was a binary variable indicating the presence of either ESBL-producing *E. coli* or *K. pneumoniae.* Our initial model contained all samples for which there was complete data on the following variables: time period (i.e., pre- or post-June 2020), infant gender, whether the infant was born in a healthcare facility, admission room, delivery mode, maternal fever at delivery, maternal fever during pregnancy, maternal use of antibiotics, maternal use of third generation cephalosporins, infant nasogastric tube, infant intravenous line, infant oxygen mask, feeding method, and whether a blood culture was taken. As fewer than 4% of the infants included in the analysis had a catheter, this variable was excluded. The diagnostic category (communicable or non-communicable illness) was excluded due to collinearity with the feeding mode. Backward model selection using the Akaike information criterion (AIC) was used to identify the best-fitting model. The final model residuals were examined using the Dharma package (Hartig, 2022). A p-value < 0.05 indicated statistical significance.

### Inclusion and exclusion criteria

We included healthcare professionals in the neonatal wards, neonates, and the caretakers of the babies who were enrolled in the study. In consultation with the neonatal ward management, all neonates present on the neonatal ward and meeting the inclusion criteria were included if parental consent was obtained. Students rotating in the neonatal care unit, visiting workers, short-term volunteers, neonates who were severely ill, preterm babies, and participants who withdrew informed consent were excluded from the study.

### Ethical clearance

Ethical permission for the study was granted by the Kilimanjaro Christian Medical University College (KCMUCo) research ethical review committee in Moshi and the Medical Research Coordinating Committee of the National Institute for Medical Research in Tanzania (NIMR-MRCC).

## Results

### Clinical and demographic characteristics of the study participant

A total of 437 neonates were screened for ESBL-producing *E. coli* and *K. pneumoniae* in their rectums. Of them, 235/437 (54%) were male. Most study participants were born in health facilities (94%, n=412) and 6% (n=25) were born at home. Of the 437 neonates with recorded data, 54.00% (236/437) were born through spontaneous vaginal delivery (SVD), 44% (193/437) were born through cesarean section (C/S), and 0.5% (2/437) were born through assisted vaginal delivery (AVD). The median gestational age and birth weight of the study participants was 38 weeks and 2.87 kg, respectively. Overall, 84% (n=369) of the neonates were on antibiotics. Some (4%, n=18) mothers were febrile during delivery with 4% (n=18) having a history of fever during delivery. Some (13%, n=55) of the mothers received antibiotics during their pregnancy, with 9% (n=40) taking third-generation cephalosporins (**Table 1**).

**Table 1:** Clinical and demographic characteristics of the study participants.

Clinical characteristic	Frequency	Proportion (%)
Gender*		
Male	235	54
Female	197	45
Neonates born at the health facility*		
Yes	412	94
Admission room*		
6A	222	51
7A	209	48
Delivery mode*		
SVD	236	54.00
C/S	193	44
AVD	2	0.5
Mother febrile at delivery*		
Yes	18	4
Fever history during delivery*		
Yes	18	4
Antibiotics use during pregnancy*		
Yes	55	13
Third-generation cephalosporin use*		
Yes	40	9
History of having catheter*		
Yes	407	93
History of having an IV line*		
Yes	398	91
History of having NGT*		
Yes	79	18.
Oxygen mask use*		
Yes	121	28
Breastfed*		
Combined+formula	126	29
Exclusively breastfed	267	61
Formula	14	3
IV Fluids	16	4
Diagnosis category*		
Communicable	126	29
Non-communicable	267	61
Neonate antibiotics*		
Yes	369	84
Blood culture ordered*		
Yes	142	32

*unrecorded data.

N= 437. IV, intravenous; NGT, nasogastric tube; AVD, assisted vaginal delivery; SVD, spontaneous vaginal delivery; C/S, cesarean section.

### Overall prevalence of ESBL-producing *E. coli* and *K. pneumoniae* species from neonatal feces, maternal hands, and neonatal cots

A total of 201 (46%) *K. pneumoniae* and 96 (22%) *E. coli* isolates were from the neonates. Out of the 297 *K. pneumoniae* and *E. coli* isolates, 294 were ESBL positive, of which, 198 (67%) were ESBL-producing *K. pneumoniae* and 96 (32%) were ESBL-producing *E. coli.* A total of 50 (11%) of the neonates had both ESBL-producing *K. pneumoniae* and *E. coli* species isolated in one sample. One (1%) *K. pneumoniae* isolate and one (1%) *E. coli* isolate were isolated from maternal hands. Furthermore, 23 (21%) *K. pneumoniae* and 4 (4%) *E. coli* isolates were from neonatal cots, while healthcare workers were not contaminated with *E. coli* or *K. pneumoniae* species. *K. pneumoniae* was the most isolated organism, followed by *E. coli*.

### Potential risk factors associated with fecal carriage of ESBL-producing *E. coli* and *K. pneumoniae* in neonates

#### Descriptive statistics

High rates of ESBL-producing *E. coli* and *K. pneumoniae* carriage were found in the neonates born at the health facility (n=282, 65%) compared to those born at home (n=25, 6%). There was a slight difference in the number of those with ESBL fecal carriage between males (n=164, 38%) and females (n=131, 30%) and in ESBL fecal carriage among neonates with a history of having an intravenous (IV) line (n= 274, 63%) compared to those without an IV line. Carriage was also greater among neonates who were exclusively breastfed compared to those who also received formula (n= 174, 40%). There was a slight difference in the number of ESBL carriages among neonates with communicable (n= 100, 23%) and non-communicable diseases (n= 174, 40%). ESBL fecal carriage was high among neonates who received antibiotics during their hospital stay (n=256, 59%) (**Table 2**).

**Table 2:** Potential risk factors associated with neonatal fecal ESBL-producing *E. coli* and *K. pneumoniae* carriage.

Characteristic		Frequency	Proportion	ESBL-producing *E. coli*	ESBL-producing *K. pneumoniae*
Gender	Male	235	54	51	113
	Female	197	45	43	88
Neonates born at the health facility	Yes	412	94	89	193
Admission room	6A	222	51	43	83
	7A	209	48	52	117
Delivery Mode	SVD	236	54	45	95
	C/S	193	44	50	103
	AVD	2	0.5	1	1
History of urinary catheter use	Yes	407	96	5	8
History of IV line use	Yes	398	91	89	185
History of NGT use	Yes	79	18	18	42
Oxygen mask use	Yes	121	28	29	66
Feeding	Combined +formula	126	29	33	67
	Exclusively breastfed	267	61	57	117
	Formula	14	3	3	9
	IV fluids	16	4	1	4
Diagnosis category	Communicable	126	51	33	67
	Non-communicable	267	48	57	117
Neonatal antibiotics	Yes	359	84	85	171

IV, intravenous; NGT, nasogastric tube; AVD, assisted vaginal delivery; SVD, spontaneous vaginal delivery; C/S, cesarean section.

### Risk factor analysis using a generalized linear model

After selecting entries with complete information (i.e., excluding entries with missing entries or “unknown”) the analysis was performed on 300 infants. The best-fitting model for the presence of ESBL-producing *E. coli* or *K. pneumoniae* (**Table 3**) showed significant associations between the presence of the organisms and the admission room and delivery mode. Associated with a significant increase in the risk of ESBL-producing *E. coli* or *K. pneumoniae* were samples from infants admitted to rooms 7 or 7A [odds ratio (OR): 1.95, confidence interval (CI): 1.21 – 3.13, p = 0.006]. Samples from infants born by vaginal delivery had a reduced risk of ESBL-producing *E. coli* or *K. pneumoniae* carriage (odds ratio: 0.57, CI: 0.35 – 0.92, p=0.023).

**Table 3:** Final model showing the risk factors associated with neonatal fecal ESBL-producing *E. coli* or *K. pneumoniae* carriage in 300 neonates.

*Predictor*	ESBL-producing *E. coli* or *K. pneumoniae*		
	*Odds ratio*	*CI*	*p*
(Intercept)	1.45	0.94–2.25	0.098
Room 7A	1.95	1.21–3.13	**0.006**
Delivery mode (standard vaginal delivery)	0.57	0.35–0.92	**0.023**
Observations	300		

The bold values represent P values.

### Antimicrobial resistance pattern for ESBL *E. coli* and *K. pneumoniae*

Overall, ESBL-producing *E. coli* and ESBL-producing *K. pneumoniae* were frequently resistant to ampicillin (n=261, 100%), trimethoprim-sulfamethoxazole (*n* = 233, 89%), gentamicin (*n* = 169, 66%), and tetracycline (*n* = 140, 54%). Only three (1%) of the ESBL-producing *E. coli* and ESBL *K. pneumoniae* isolates were resistant to meropenem.

### Multi-drug resistance in ESBL-producing *E. coli* and *K. pneumoniae*

Overall, 82% (253/310) of the ESBL-producing *E*. *coli* and *K*. *pneumoniae* isolates were multidrug-resistant (MDR, resistance to ≥3 antibiotics). Among the total isolates for each species, MDR *E*. *coli* comprised 75% (62/83) and MDR *K*. *pneumonia* 86% (191/222). Only one ESBL-producing *K*. *pneumoniae* isolate was resistant to all antibiotics including meropenem.

## Discussion

ESBL-producing *E. coli* and *K. pneumoniae* are major multidrug-resistant bacteria that are known to cause serious hospital and community-acquired infections worldwide. *E. coli* and *Klebsiella* infections are usually treated with common antibiotics, such as penicillin and cephalosporin. However, when these bacteria produce ESBLs, they become resistant to these antibiotics and this significantly limits treatment options, contributing to the progression of infection and in some cases death (Kumburu et al., 2019). In the current study, we assessed the burden, risk factors, and antimicrobial susceptibility patterns associated with the carriage of ESBL-producing *E. coli* and *K. pneumoniae* among neonates, and in their surroundings, at a referral hospital in north-eastern Tanzania.

In our study, we found a high burden of ESBL-producing *K. pneumoniae* and *E. coli* among neonates and the environment, while resistance to ampicillin and gentamicin was also frequent. This is similar to other findings in other studies in Africa (Herindrainy et al., 2011; Isendahl et al., 2012; Farra et al., 2016; Karanika et al., 2016; Tellevik et al., 2016; Sanneh et al., 2018; Letara et al., 2021).

In our study, the overall prevalence of ESBL-producing *K. pneumoniae* and *E. coli* fecal carriage among neonates was 67%. The overall prevalence of ESBL-producing *K. pneumoniae* and *E. coli* fecal carriage among neonates was 67% and 32%, respectively. This prevalence is higher compared to the observations from other studies (Tellevik et al., 2016; Letara et al., 2021).

This difference could be due to differences in study participants and risk factors associated with carriage. This finding aligns with the results of other studies conducted in the Mwanza region of Tanzania, where a notably high prevalence of ESBL-producing *E. coli* and *K. pneumoniae* was observed among street children. These studies have highlighted the issue of antimicrobial resistance in this vulnerable population, emphasizing the increased risk of infections caused by these drug-resistant pathogens (Moremi et al., 2017).

In total, 5% of maternal hands and 22% of neonatal cots were found to be contaminated with ESBL-producing *E. coli* and *K. pneumoniae*. This is consistent with a study conducted in the Mwanza region, where maternal hands and neonatal cots were also found to be contaminated with multidrug-resistant Gram-negative bacteria (Silago et al., 2020).

This could be due to less emphasis on the importance of hand washing and frequent decontamination of neonatal cots, which may result in the acquisition of ESBLs; therefore, these surfaces may serve as reservoirs for transmission, highlighting the importance of regular disinfection and hand hygiene within the neonatal ward. Healthcare workers were not contaminated with either *E. coli* or *K. pneumoniae*. All healthcare workers involved in the study were observed to have carried portable hand sanitizers during their daily routine and the results suggest a proper adherence to hand decontamination. ESBL-producing *K. pneumoniae* (67%) was predominantly isolated from the neonates followed by ESBL-producing *E. coli* (32%), in contrast with studies that reported *E. coli* to be predominant (Kumburu et al., 2019). This calls for more research in this area.

In this study, we assessed the risk factors associated with fecal carriage of ESBL-producing E. coli and K. pneumoniae in neonates. High rates of ESBL-producing E. coli and K. pneumoniae carriage were found in those born at the health facility (n=282, 65%) compared to those born at home (n=25, 6%). There was a slightly significant difference in the number of those with ESBL fecal carriage between males (n=164,38%) and females (n=131, 30%). ESBL fecal carriage was slightly higher among neonates with a history of having an IV line (n= 274, 63%) and neonates who were exclusively breastfed (n= 174, 40%). There was no significant difference in ESBL carriage among neonates with communicable (n= 100, 23%) and non-communicable diseases (n= 174, 40%). ESBL fecal carriage was high among neonates who were on antibiotics during their hospital stay (n=256, 59%) (**Table 2**). The results suggest that the admission room is a significant risk factor for the carriage of ESBL-producing *E. coli* or *K. pneumoniae* in neonates, with an increased likelihood of carriage observed in neonates admitted to the hospital (OR = 1.95, p = 0.006). Additionally, delivery mode appears to influence the risk, with vaginal delivery being associated with a reduced risk of carriage (OR = 0.57, p = 0.023), indicating that neonates born via vaginal delivery may have a lower likelihood of acquiring these resistant pathogens compared to those delivered by other methods and, therefore, optimizing hospital room hygiene and reconsidering delivery practices, especially in the case of delivering neonates, are recommended. In this study, ESBL-producing *E*. *coli* and *K. pneumonia* isolates were frequently resistant to ampicillin (n=261, 100%), trimethoprim-sulfamethoxazole (*n* = 233, 89%), gentamicin (*n* = 169, 66%), and tetracycline (*n* = 140, 54%). This is in line with other studies from Tanzania, which reported frequent resistance to tetracycline (100%), trimethoprim-sulfamethoxazole (97%), ciprofloxacin (69%), and gentamicin (44%) (Moremi et al., 2017). In Madagascar, frequent resistance was reported to trimethoprim-sulfamethoxazole (91%), gentamicin (76%), and ciprofloxacin (50%) (Andriatahina et al., 2010). In Spain, resistance to nalidixic acid (65%), ciprofloxacin (32%), levofloxacin (32%), and trimethoprim-sulfamethoxazole (41%) were frequent in ESBL-producing *K. pneumoniae* (Fernández-Reyes et al., 2014). These findings are consistent with another study conducted in the Mwanza region of Tanzania, where resistance to ampicillin and gentamicin was also reported (Marando et al., 2018). This similarity could be due to the presence of resistance genes. Our study also observed high frequencies of multidrug resistance in ESBL-producing *E. coli* and *K. pneumoniae*. Overall, 82% (253/310) of the ESBL-producing *E*. *coli* and *K*. *pneumoniae* isolates were MDR, defined as resistance to ≥3 antibiotics. Among the total isolates for each species, MDR *E*. *coli* comprised 75% (62/83) and MDR *K*. *pneumonia* 86% (191/222). Only one ESBL-producing *K*. *pneumoniae* isolate was resistant to all antibiotics, including meropenem. This highlights the need for antimicrobial stewardship in neonatal wards to limit the use of broad-spectrum antibiotics. It will be of interest to study the molecular characterization of ESBL-producing *E. coli* and *K. pneumoniae* to determine the role of resistance genes and their mechanisms in accelerating drug resistance. Meanwhile, to ensure the effectiveness of infection control measures and prevent the transmission of resistant bacteria, active screening on admission to a specific ward could effectively limit and prevent the spread of resistant bacteria. In addition, antimicrobial stewardship should promote the appropriate use of antimicrobials (including antibiotics), which would improve patient outcomes, reduce antimicrobial resistance, and decrease the spread of infections caused by multidrug-resistant organisms. Our study has several limitations including its cross-sectional design, which allows for the collection of data at a single point in time and is therefore unable to examine changes over time. There is a need for more in-depth research in the future. Furthermore, the study was conducted at only one site which may not represent the practices, infection rates, or population demographics of other healthcare settings, and the single-center design limits the external validity.

## Conclusion

Our findings indicated a high burden of ESBL-producing bacteria colonizing neonates, maternal hands, and neonatal cots. In addition, resistance to first-line antibiotics, ampicillin and gentamicin, was also frequent. To ensure the effectiveness of infection control measures and prevent the transmission of resistant bacteria, active screening on admission to a specific ward could limit and prevent the spread of resistant bacteria, especially by implementing routine screening for ESBL-producing bacteria upon neonatal admission, strengthening hand hygiene protocols, and frequently disinfecting neonatal cots and surfaces. The establishment of microbiological methods to detect resistant bacteria could help guide empiric antimicrobial chemotherapy.

## Data Availability

The raw data supporting the conclusions of this article will be made available by the authors, without undue reservation.
